# Micropore-Confined Organic Solid for a High-Rate and Durable Electrode

**DOI:** 10.1021/acsami.5c11604

**Published:** 2025-07-28

**Authors:** Kaiya Nakasone, Showa Kitajima, Hitoshi Kasai, Kouki Oka, Daisuke Takimoto

**Affiliations:** 1 Graduate School of Science and Engineering, 26428 University of the Ryukyus, Nishihara, Okinawa 903-0213, Japan; 2 Institute of Multidisciplinary Research for Advanced Materials, 13101 Tohoku University, 2-1-1 Katahira, Aoba-ku, Sendai, Miyagi 980-8577, Japan; 3 Carbon Recycling Energy Research Center, Ibaraki University, 4-12-1, Nakanarusawacho, Hitachi, Ibaraki 316-8511, Japan; 4 Deuterium Science Research Unit, Center for the Promotion of Interdisciplinary Education and Research, Kyoto University, Yoshida, Sakyo-ku, Kyoto 606-8501, Japan; 5 Faculty of Science, 26428 University of the Ryukyus, Nishihara, Okinawa 903-0213, Japan

**Keywords:** anthraquinone, micropore-confined organic solid, rechargeable organic-based aqueous air battery, high-rate and durable electrode, electrochemical impedance spectroscopy

## Abstract

The redox reactions of solid-state anthraquinone (AQ) confined within the micropores of activated carbon (AC) were investigated. A thin layer of AQ solid was adsorbed into the micropores of AC without inhibiting the formation of the electrical double layer. The AQ solid exhibited pseudocapacitive behavior and achieved 100% of its theoretical charge capacity (926 C g_‑AQ_^–1^). Electrochemical impedance spectroscopy revealed that the capacitor response time of the AQ solid was shorter than those of the molecules dissolved in the electrolyte. This indicates that a direct charge transfer occurred from the carbon surface to the AQ solid. Regarding rechargeable aqueous air battery performance, the AQ solid demonstrated a discharge capacity of 257 mAh g_‑AQ_^–1^ (99% of theoretical capacity) and a Coulombic efficiency of 99%. Both the cycling stability and rate performance of the AQ solid were superior compared with previously reported anode materials for rechargeable aqueous air batteries.

## Graphical abstract

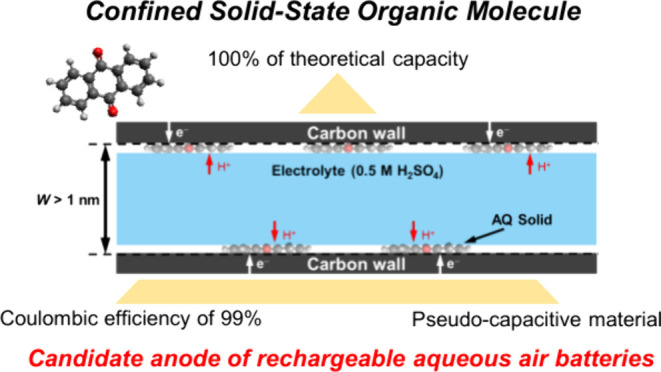

## Introduction

Developing energy-storage devices with improved capabilities has been extensively studied to achieve sustainable societies.^1,2^ Rechargeable aqueous air batteries are an attractive energy-storage application and offer safer alternatives to Li-ion batteries.^3–5^ Aqueous batteries are an attractive energy-storage device due to their unique merits, such as being compared to organic solvent-based electrolytes, high ionic conductivity, nonflammability, less sensitivity to ambient air, and environmental friendliness. Metal-based anodes have been widely investigated, but the hydrogen evolution reaction accelerates self-discharge. To overcome this issue, redox active molecules are considered a potential candidate for anode material because of their low activity for hydrogen evolution reaction, abundant, readily available building blocks (H, C, and O), and tunable properties.

Quinone-based aromatic compounds are promising candidates for electrode materials in energy-storage devices.^6,7^ However, their practical application is limited by challenges such as redox reversibility, rate performance, capacity, and durability. Various strategies, including chemical^8–10^ and physical adsorption,^11–13^ have been reported to enhance redox reversibility. Despite these efforts, the electrodes exhibit peak potential separations of Δ*E*_p_ > 57/*n* mV at 25 °C (*n* = number of electrons transferred), as seen by cyclic voltammetry. This likely arises from inherent charge-transfer resistance under ambient conditions, which broadens Δ*E*_p_.^14,15^

The confinement of quinone-based molecules within subnanometer micropores of activated carbon enhances redox reversibility, often resulting in zero peak separation in their voltammograms.^16–19^ In addition, confined electrodes exhibit superior rate performance compared with their nonconfined counterparts. X-ray scattering and hybrid reverse Monte Carlo simulations revealed strong adsorption of confined quinone on carbon surfaces, with an estimated molecule-to-carbon surface distance of approximately 0.3 nm. According to the Nernst equation, this proximity facilitates the capacitive redox behavior. However, such behavior is typically observed only at low-molecule loadings on activated carbon (typically less than 10 wt %), as near-saturation loading levels lead to dissolution into the electrolyte,^19^ highlighting a trade-off between redox reversibility and capacity. Furthermore, the durability of these confined systems remains limited; degradation of the electrodes is attributed to molecular decomposition via the Michael reaction.^17^

In this study, we developed a nonpolar AQ solid confined within the micropores of activated carbon (20 wt % AQ), which exhibited excellent redox reversibility and high capacity (257 mAh g^–1^ corresponding to 100% of the theoretical value). The thin layer of AQ solid was adsorbed into the micropores of AC without disrupting the formation of the electrical double layer (EDL). The AQ solid exhibited ideal reversible redox behavior with capacitive characteristics, classifying it as a pseudocapacitive material. As a proof of concept, a rechargeable organic-based aqueous air battery was fabricated, yielding a high discharge capacity of 257 mAh g_‑AQ_^–1^, long-lifetime of >100 cycles, and notably high-rate performance (up to 60C rate).

## Experimental Section

### Reagents

Anthraquinone (AQ; Tokyo Chemical Industry Co. Ltd., Japan) and sodium anthraquinone-2-sulfonate monohydrate (AQS; Tokyo Chemical Industry Co. Ltd., Japan) were used as the electroactive molecule. Micropore-rich KOH-activated carbon (AC; MSP-20X, Kansai Coke and Chemicals Co., Ltd.) was used as the carbonaceous material of the electrodes. Sulfuric acid (H_2_SO_4_; super special grade, FUJIFILM Wako Pure Chemical Corporation) was used as the electrolyte. *N*,*N*-Dimethylformamide (DMF; special grade) was purchased from FUJIFILM Wako Pure Chemical Corporation. Milli-Q ultrapure water (>18.2 MΩ cm, Merck Millipore, USA) was used in all experiments. All materials were used without further purification.

### Preparation of Electrode Materials

AQ was dissolved into DMF, a 0.1 mM AQ solution was prepared, and AC (200 mg) was dispersed in the solution (100 mL). The suspension was magnetically stirred at room temperature for 24 h, collected by suction filtration, and dried at 333 K for 24 h. The loading of AQ on AC was controlled by changing the concentration of the AQ solution. The amount of adsorbed AQ on AC was determined by ultraviolet–visible (UV–vis) spectroscopy (V-660, 190–600 nm, 400 nm min^–1^, JASCO) (Figure S1). The loading of AQ on AC were 1.0, 4.9, 8.3, and 19.0 wt %, denoted as AQ(1)/AC, AQ(5)/AC, AQ(10)/AC, and AQ(20)/AC, respectively.

### Characterization of Electrode Materials

The morphology of the samples was observed by scanning electron microscopy (SEM; TM4000PlusII, accelerating voltage of 5 kV, Hitachi High-Tech Corporation). X-ray diffraction (XRD) was conducted using a RINT ULTIMA (Rigaku) with Cu Kα radiation (λ = 1.5418 Å) operating at 15 kV and 40 mA to characterize the crystallinity of the samples. Adsorption and desorption isotherms were obtained using a volumetric apparatus (BELSORP-MAX, MicrotracBEL) with liquid N_2_ gas as the adsorbate at 77 K. Prior to the measurements, the adsorbed water was carefully removed by vacuum drying at 423 K for 3 h. The Brunauer–Emmett–Teller equation was used to calculate the specific surface area in a *p*/*p*_0_ range of 0.01–0.05 to prevent overestimation of the surface area. Nonlocal density functional theory calculations were used to determine the specific surface area and pore width distributions. Fourier transform infrared (FT-IR) spectroscopy (FT-IR-6100, JASCO) and Raman spectroscopy (XploRA PLUS, HORIBA) were used to characterize the chemical state and microstructure of the materials. Single-Crystal XRD analysis of AQ was performed on a Rigaku Saturn724+ diffractometer using graphite monochromated Mo Kα radiation (λ = 0.71075 Å; Table S1). Molecular dynamics simulation results were obtained from the Winmostar V9 (X-Ability Co. Ltd., Japan) using DFT calculations with the B3LYP functional and 6-31G basis set. Intervals of sections and radius of the solvent were 0.02000 and 2.00000 Å, respectively. The total density of the molecule was 0.182 nm^3^ and the *x–y* plane area was 0.689 nm^2^.

### Electrochemical Measurements

Electrochemical measurements were conducted using a potentiostat/galvanostat (HZ – 7000, MEIDEN HOKUTO) with a three-electrode system consisting of Pt mesh counter and Ag/AgCl (KCl saturated) reference electrodes. Electrode potentials were referred to as the reversible hydrogen electrode (RHE) potential scale and calculated using the following equation:

$$E\mathrm{vs}.\mathrm{RHE}=E\mathrm{vs}.\mathrm{Ag}/\mathrm{AgCl}+(224-T)\times \frac{\partial T}{\partial E}-\frac{RT}{nF}\times \mathrm{pH}\times 2.303$$ S1

where *R* is the gas constant (8.314 J K^–1^ mol^–1^), *T* is the temperature (K), *n* is the number of electrons involved in the electrode reaction, *F* is the Faraday constant (96,485 C mol^–1^), and ∂*T*/∂*E* is 0.0010 K V^–1^. The electrode material powder was dispersed in Milli-Q ultrapure water (2 g L^–1^), and an aliquot of 10 μL was deposited onto a mirror-polished glassy carbon electrode (3 mm in diameter, *A* = 0.07 cm^2^). A 1 wt % Nafion solution (5 μL) was then cast to affix the electrode material to the glassy carbon, resulting in a loading of 20 μg_‑active material_ cm^–2^. Potential cycling was conducted prior to electrochemical measurements in a deaerated 0.5 M H_2_SO_4_ (*E* = 0–1.0 V vs RHE, 298 K, 200 mV s^–1^, 100 cycles). Cyclic voltammetry was conducted between 0 and 1.0 V vs RHE at scan rates of 5–500 mV s^–1^. The cycle stability test was conducted between 0 and 1.0 V vs RHE at scan rates of 50 mV s^–1^ for 1000 cycles (298 K). Electrochemical impedance spectroscopy (EIS) was carried out in the same type of electrochemical cell as that for cyclic voltammetry (25 °C). Impedance measurements were conducted using fresh electrodes (0.1, 0.3, 0.5, 0.7, and 0.9 V vs RHE) by sweeping frequencies from 50 kHz to 10 mHz at an amplitude of 5 mV. The impedance data are normalized to the geometric surface area of the glassy carbon electrode (0.07 cm^2^).

### Rechargeable Air Battery Evaluation

A tailor-made glass cell (20 cm^3^ electrolyte, Watanabe Kagaku) was employed as the electrochemical cell. AQ(20)/AC and poly­(vinylidene fluoride) were mixed in *N*-methyl-2-pyrrolidone with a mass ratio of 30:70. The obtained slurry was casted onto a glassy carbon (1 cm^2^). The AQ(20)/AC was used as the anode (0.8 mg_‑material_ cm^–2^ (0.16 mg_‑AQ_ cm^–2^)), and the Pt nanoparticle-supported carbon catalyst (40 wt % Pt/C, Fuel Cell Earth) was used as the cathode (11.1 mg cm^–2^). Both the anode and cathode sections were filled with 0.5 M H_2_SO_4_ aqueous solution. The anode-side was deaerated by nitrogen, and the cathode-side was open to the air. Galvanostatic charge/discharge testing was conducted with a voltage window of 0–1.7 V vs cathode at 10–60C (1C = 257 mA g_–AQ_^–1^) using a potentiostat system (HZ–7000).

## Results and Discussion

### Characterization of Physical Properties of AQ/AC

The adsorption isotherm was investigated by UV–vis spectroscopy to evaluate the presence and amount of adsorbed AQ on AC (see Supporting Information, Figure S1). AQ reached saturation adsorption within 15 min (Figure S1a). The adsorption model of AQ on AC was determined using the curve-fitting analysis.^20^ According to the curve-fitting analysis with the normalized standard deviation (Δ*y* (%)),^21^ the adsorption model of AQ on AC followed the Langmuir adsorption model. In addition, the equilibrium adsorption of AQ on AC was 19.0 wt % from the Langmuir equation (denoted as AQ(20)/AC). The morphology of the samples was investigated by using scanning electron microscopy (SEM). The morphologies of AC and AQ were markedly different; AQ crystals had lateral dimensions of several hundred micrometers, approximately 10 times larger than that of AC (Figure 1A,B). The morphology of AQ(20)/AC closely resembled that of AC (Figure 1C), suggesting that AQ did not form a plate-like crystal structure independently. X-ray diffraction peaks corresponding to AQ were not observed from the diffraction patterns of AQ(20)/AC (Figure S2), indicating the absence of AQ crystals on the outer surface of AC. Moreover, Fourier transform infrared and Raman spectroscopy revealed that the crystallinity and surface functional groups of AC remained unaffected by AQ adsorption process (Figures S3 and S4 and Table S2).

**Figure 1:**
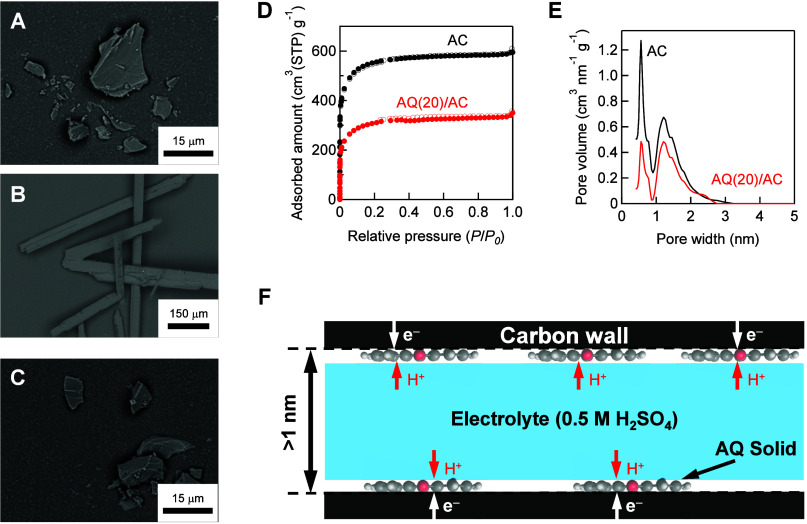
Morphology and characterization of the pore structure of the materials. Typical SEM images of (A) AC, (B) AQ crystal, and (C) AQ(20)/AC. (D) N_2_ adsorption–desorption isotherms at 77 K of AC (black) and AQ(20)/AC (red). Closed circles and open squares represent adsorption and desorption, respectively. (E) Pore width distribution profiles of AC (black) and AQ(20)/AC (red) derived using nonlocal density functional theory calculations. (F) Schematic of AQ adsorption sites into micropore of AC.

The adsorption sites of AQ on AC were characterized by using N_2_ adsorption/desorption isotherms. The amount of adsorbed nitrogen decreased after AQ adsorption (Figure 1D); the specific surface area of AQ(20)/AC was 1106 m^2^g^–1^ (Table S3), which was half that of AC (2002 m^2^ g^–1^). Based on the area of AQ (0.689 nm^2^ obtained from DFT calculation), the coverage of AQ on AC was 23.3%. The cumulative pore volume of AQ(20)/AC was 0.54 cm^3^ g^–1^, lower than AC’s 0.94 cm^3^ g^–1^ (Figure 1E). Based on the density of AQ (0.67 cm^3^ g^–1^), micropore volume of AC (0.83 cm^3^ g^–1^), and mass of AQ and AC for AQ(20)/AC, 18.9% of AC’s micropore volume was lost after AQ adsorption. The calculated pore volume of AQ(20)/AC (0.54 cm^3^ g^–1^) matches that obtained from the N_2_ adsorption/desorption results. If AQ were only adsorbed at the entrances of AC’s pores, the measured pore volume could be significantly lower. The average micropore width of AQ(20)/AC was 0.91 nm, similar to AC’s 0.90 nm. If AQ was densely adsorbed deep within micropores smaller than 1 nm, the cumulative pore volume would be lower than that of AC while the average micropore width would increase. These results indicate that AQ is uniformly adsorbed within AC’s micropores, suggesting that the thickness of the adsorbed AQ is likely a single layer.

### Redox Reactions of a Micropore-Confined AQ Solid

The redox reactions of AQ/ACs were investigated by using a three-electrode system. All samples exhibited a rectangular cyclic voltammogram, indicative of an electrical double-layer formation on the electrode materials (Figure 2A and Figure S5). In AQ/AC samples, sharp peaks appeared at 0.1 V vs RHE, corresponding to the redox reaction of AQ. The peak current increased with an increasing AQ content on AC (Figure S5). The redox peak potential separation (Δ*E*_p_) of AQ(20)/AC was 12 mV, indicating the remarkable reversibility of the redox reaction. This Δ*E*_p_ value was significantly smaller than those reported for conventional physical and chemical adsorption methods (Table S4).^8,12,13,22–28^ The capacitance of AQ(20)/AC decreased with the scan rate, displaying a capacitance loss of 43%, likely due to *iR* losses from inter- and/or intraparticle resistance in AQ(20)/AC (discussed later). The scan rate dependence of AQ/AC was slightly lower than that of AC (Figure 2B and Table S5). Notably, the redox potential of AQ/ACs was not clearly observed at high scan rates (above 250 mV s^–^^1^, Figure S6), which may have led to an underestimation of the scan rate performance. In addition, hydrogen evolution reaction was observed on expanding the potential window, thus limiting the accurate evaluation of scan rate performance.

**Figure 2:**
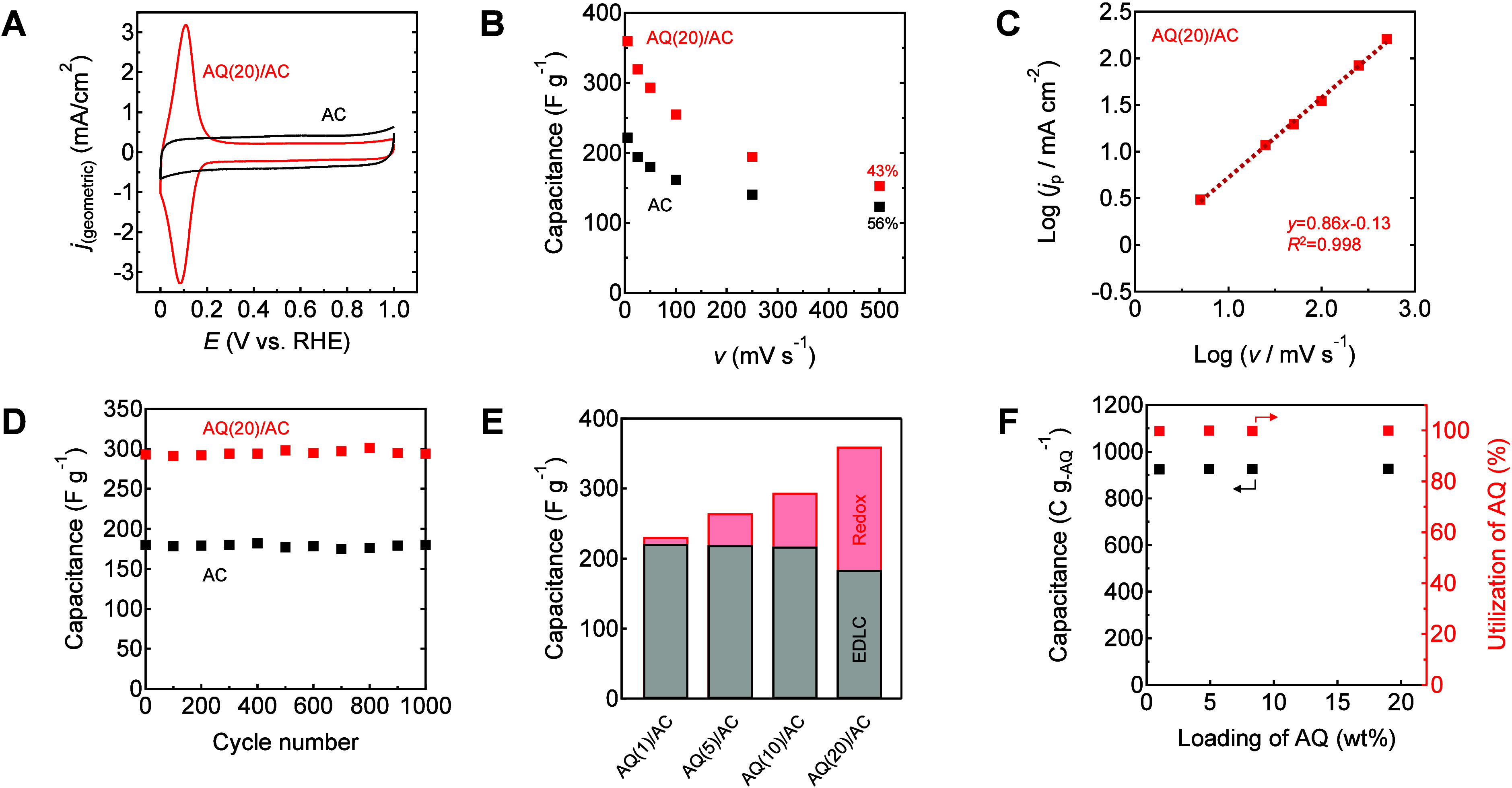
Electrochemical Characterization of the AQ solid adsorbed on AC. (A) Cyclic voltammograms of AC and AQ(20)/AC at a scan rate of 5 mV s^–1^ in deaerated 0.5 M H_2_SO_4_ (298 K). (B) Scan rate dependences of AC and AQ(20)/AC. (C) *b*-values (slopes) derived from the current peaks (*j*_p_ = *av* ^*b*^) of the cyclic voltammograms of AQ(20)/AC. (D) Capacitance retention of AC and AQ(20)/AC as a function of cycle number. (E) Separation of capacitance to electrical double-layer charge and redox charge of AQ/ACs. (F) Capacity of AQ (black: left axis) and AQ utilization (red: right axis) as functions of AQ loading.

The contributions to the current from mass-transfer- and adsorption-controlled processes were distinguished via the *b*-value analysis.^29^ The current response obeys the power–law relationship:

$$i=av^{b}$$ 1

where *i* is the measured current, *v* is the scan rate, and *a* and *b* are fitting parameters. The value of *b* can be determined from the slope of a log­(*i*) vs log­(*v*) plot (Figure S7). At 0.1 V, that is, the half-wave potential of the redox reaction of AQ, the *b* value of AQ(20)/AC was 0.86 (Figure 2C), indicating a predominantly capacitive contribution governed by surface-controlled kinetics. This was further corroborated by the linear dependence of both the anodic (*i*_pa_) and cathodic (*i*_pc_) peak currents on the scan rate (*v* = 5–500 mV s^–^^1^) (Figure S8).

It should be noted that the reaction system was not adsorption-controlled but rather diffusion-controlled when anthraquinone-2,6-disulfonic acid (AQDS) and sodium anthraquinone-2-sulfonate monohydrate (AQS) were adsorbed on AC, forming AQDS/AC and AQS/AC composites, respectively (Figure S9). This behavior is attributed to the dissolution of these molecules from the carbon surface owing to their high aqueous solubility (62 mg mL^–1^ for AQDS and 9.8 mg mL^–1^ for AQS^30^). In contrast, owing to its low aqueous solubility (<1 mg mL^–1^), AQ remains solid within the micropores resulting in high stability during electrochemical cycles (Figure 2D). These results suggest that the faradaic reaction occurs directly on the AQ solid adsorbed on the AC surface, exhibiting capacitive behavior; thus, AQ/AC can be classified as pseudocapacitive material.

It can be concluded that the electrical double-layer charge of AC was not affected by AQ adsorption. The electrical double-layer charge for AQ/ACs ranged from 196 to 208 F g^–1^, which is comparable to that of AC (221 F g^–1^). This indicates that the formation of EDL was not inhibited by AQ solid adsorption, a conclusion further supported by N_2_ adsorption–desorption isotherms. This also suggests the formation of an inner Helmholtz plane on the AQ solid surface.

The specific capacitance, normalized to the mass of the active material (AQ and AC), increased with an increasing AQ loading (Figure 2E). For AQ(20)/AC, the value reached 360 F g^–1^, which is 1.6 times higher than that of AC (221 F g^–1^), primarily due to the redox charge contribution of AQ (Table S6). The specific capacitance normalized to the AQ mass for all AQ/AC samples was 926 C g_‑AQ_^–1^, matching the theoretical charge of AQ and indicating 100% utilization (Figure 2F and Table S7). This suggests that all adsorbed AQ solids were electrochemically active, which was ascribed to the facile charge-transfer reaction between AQ and AC when the AQ solid layer was sufficiently thin.

### Electrochemical Impedance Spectroscopy

To investigate the kinetics of the charge storage process involving a two-proton/two-electron transfer, EIS was conducted under constant potential conditions (0.1–0.9 V vs RHE, Figures S10 and S11). The redox response of AQS dissolved in the electrolyte was also characterized as a control. The Nyquist plots for various samples at 0.1 V vs RHE, along with the corresponding complex-plane impedance plots, exhibited a near-vertical line along the imaginary axis (Z″) across all potentials, characteristic of an ideally polarizable electrode. The solution resistance (*R*_sol_) remained nearly constant across all samples (∼0.6 Ω cm^2^). Because the dissolved AQS concentration was 1 mM in 0.5 M H_2_SO_4_, the *R*_sol_ was not largely affected by the presence of AQS. For the AQ/AC, the AQ solid was adsorbed on the carbon surface; therefore, the *R*_sol_ for all samples was constant. In the case of AC, no clear semicircle was observed, likely due to the dominant contribution of the electrical double-layer capacitance (*C*_dl_). In contrast, the semicircle was observed from dissolved AQS and solid AQ, which can be attributed to the charge-transfer reaction. The charge-transfer resistance (*R*_ct_) of solid AQ was 1.7 Ω cm^2^, similar to or slightly larger than that of dissolved AQS (1.2 Ω cm^2^), indicating that *R*_ct_ was not primarily affected by the dissolved or solid states of molecules. In both states, the charge-transfer process is initiated with the adsorption of molecules on the carbon surface. Furthermore, anthraquinone-based molecules prefer to adsorb with π–π stacking onto the carbon surface. The N_2_ adsorption–desorption results indicate that solid AQ is adsorbed in a monolayer on the carbon surface. Thus, in this study, the *R*_ct_ was not primarily affected by whether AQ was dissolved or solid.

The transition frequency (*f*_t_) is defined as the frequency at which the system undergoes transitions from mass-transfer-controlled to charge-transfer-controlled behavior, serving as an indicator of redox kinetics. The admittance spectra at 0.1 V vs RHE (Figure 3B) showed that for AQ solid, *f*_t_ = 2.5 kHz, which is comparable to that of dissolved AQS (5.0 kHz), indicating similar redox kinetics. Assuming a simplified equivalent circuit consisting of *R*_sol_, *R*_ct_, and *C*_dl_ (Figure S12), the peak frequence (*f*′_max_) of the admittance spectra can be calculated using the following equation:

$$f_{\max}^{\prime }=\frac{1}{2\pi (R_{\mathrm{ct}}C_{\mathrm{dl}})}\left(\frac{R_{\mathrm{ct}}}{R_{\mathrm{sol}}}+1\right)$$ 2

**Figure 3:**
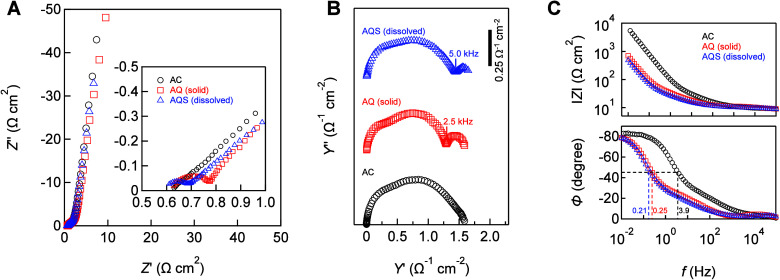
Characterization of the kinetics of the charge storage process using electrochemical impedance spectroscopy. (A) Nyquist plots, (B) admittance spectra, and (C) frequency dependence of the impedance magnitude |*Z*| and phase angle ϕ at 0.1 V vs RHE of AC (black circles), AQ(20)/AC (red squares), and 1 mM AQS+0.5 M H_2_SO_4_ (blue triangles).

Since *R*_sol_ remains constant across all samples in the Nyquist plots, *f*′_max_ depends solely on *R*_ct_ and *C*_dl_. The *f*′_max_ of AC remained unaffected by the applied potential (*f*′_max_ = 0.2 Ω^–1^ cm^–2^), suggesting that the sum of *R*_ct_ and *C*_dl_ was constant across the potential range. For both solid AQ and dissolved AQS, *f*′_max_ was small at 0.1 V vs RHE (near redox reaction potential), whereas above 0.3 V vs RHE (in the *C*_dl_ dominated potential region), the value remained fairly constant, indicating an increase in *R*_ct_ at the redox reaction potential.

The frequency dependence of the impedance magnitude (|*Z*|) and phase angle (ϕ) at 0.1 V vs RHE is shown in Figure 3C. In the high-frequency region, |*Z*| and ϕ values of AC remained unchanged between 0.1 and 0.9 V vs RHE (Figure S10), which can be attributed to the dominance of the EDL formation. In contrast, for both solid AQ and dissolved AQS, notable changes in |*Z*| and ϕ were observed at 0.1 V vs RHE (Figure 3C), particularly in the medium- and low-frequency regions. The frequency at which φ = −45°(*f*_φ=__–__45_) is indicative of the capacitive response frequency of the system. For solid AQ, *f*_φ=__–__45_ was 0.25 Hz, comparable to that of dissolved AQS (0.21 Hz), with corresponding response times of 4 and 5 s, respectively. Based on the above discussion, the adsorption of AQ solid on the carbon surface did not largely affect the charge-transfer kinetics as compared to that of dissolved AQS.

Proton-coupled electron transfer (PCET) in AQ solid was observed through its characteristic two-proton/two-electron redox reaction (Figure S13). Previous studies have shown that the dynamics of proton–electron-coupled reactions in quinone-based solids can be modulated by external response in the presence of an electric field.^31–33^ In this study, solid AQ experienced an electric field in the micropores of AC, resulting in changes in the dynamics of its PCET process. These changes can be clarified via in situ measurements of proton dynamics and theoretical calculation techniques.

### Charge–Discharge Performance of Rechargeable Organic-Based Aqueous Air Battery

Finally, a rechargeable organic-based aqueous air battery was fabricated using the AQ(20)/AC composite as the anode and a Pt/C catalyst as the cathode (Figure S14). The charge–discharge curves at the 10C rate confirm that the battery exhibits both chargeability and dischargeability, achieving a high Coulombic efficiency of 99% (Figure 4A). The discharge capacity of the active component of the anode was 257 mAh g_‑AQ_^–1^ (99% of its theoretical capacity), indicating nearly complete contribution of all of the molecules in charging and discharging. Even at a high rate of 60C rate, the discharge capacity remained at 257 mAh g_-AQ_^–1^ (Figure 4B), demonstrating an excellent rate capability. Furthermore, the discharge capacity remained >99% even after 100 cycles (Figure 4A; inset), indicating a high cyclability. Performance comparison of rechargeable organic-based aqueous air batteries (Figure 4C) shows that the present system exhibits the highest capacity retention after 100 cycles as well as the highest ratio of discharge capacity to theoretical capacity compared with conventional rechargeable organic-based aqueous air batteries (Table S8).^34–41^

**Figure 4:**
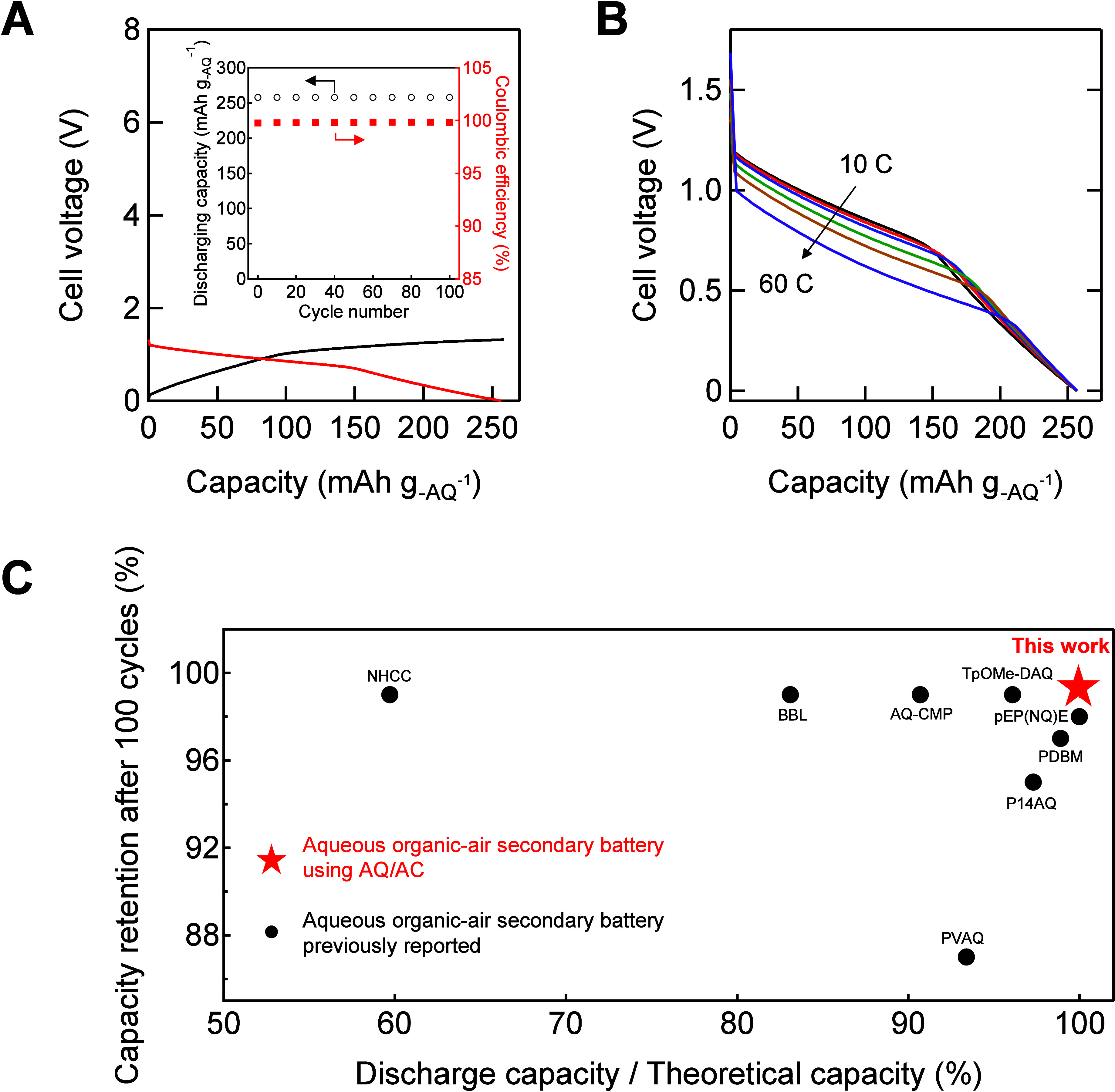
Charge–discharge performance of organic solid anode for rechargeable battery. (A) Charge (black) and discharge (red) curves of the rechargeable organic-based aqueous air battery at 10C rate (theoretical capacity = 257 mAh g_‑AQ_^–1^) and discharge capacity and Coulombic efficiency as a function of cycle number. (B) Discharge curves at 10, 15, 20, 30, 40, and 60C rate. (C) Performance summary of previously reported rechargeable organic-based aqueous air batteries.^34–41^ Details are given in Table S8.

## Conclusions

In conclusion, we investigated the redox behavior of solid AQ confined within the micropores of AC and demonstrated its application as an anode in a rechargeable battery. A thin layer of solid AQ was adsorbed into the micropores without inhibiting EDL formation. The redox process exhibited ideal reversibility, with capacitive characteristics characterizing solid AQ as a pseudocapacitive material. The measured redox charge of 926 C g_‑AQ_^–1^ corresponded to 100% utilization of AQ. EIS revealed a faster capacitor response time for solid AQ than that for dissolved AQS, indicating a direct charge transfer from the carbon surface to solid AQ. As a rechargeable battery, solid AQ achieved a discharge capacity of 257 mAh g_‑AQ_^–1^ (99% of its theoretical capacity) and a Coulombic efficiency of 99%. Moreover, its cycling stability and rate performance outperformed those of previously reported systems. This study provides the applicability of solid-state organic electrodes. Further capacity improvement will be necessary for practical applications, and fundamental studies using in situ analysis and theoretical calculations will be required to obtain insight into the redox mechanisms of solid-state organics.

## Supplementary Material



## Notes

Additional experimental details, schematic of battery setup, and summary of previous results (PDF)
