# Supplementary material for: Micropore-Confined Organic Solid for a High-Rate and Durable Electrode
Source: ACS Appl Mater Interfaces. 2025 Jul 28;17(31):44631–8. doi: 10.1021/acsami.5c11604 (PMC12333612; doi:10.1021/acsami.5c11604)
Supplement: Supplementary file 1 [file am5c11604_si_001.pdf]

Supporting Information

Micropore-Confined Organic Solid for High-Rate and Durable Electrode

Kaiya Nakasone[1†], Showa Kitajima[2†], Hitoshi Kasai[2], Kouki Oka[2,3,4] and Daisuke Takimoto*[5]*

[1]Graduate School of Science and Engineering, University of the Ryukyus, Nishihara, Okinawa 903-0213, Japan.

[2]Institute of Multidisciplinary Research for Advanced Materials, Tohoku University, 2-1-1 Katahira, Aoba-ku, Sendai, Miyagi 980-8577, Japan

[3]Carbon Recycling Energy Research Center, Ibaraki University 4-12-1, Nakanarusawacho, Hitachi, Ibaraki 316-8511, Japan

[4]Deuterium Science Research Unit, Center for the Promotion of Interdisciplinary Education and Research, Kyoto University, Yoshida, Sakyo-ku, Kyoto 606-8501, Japan

[5]Faculty of Science, University of the Ryukyus, Nishihara, Okinawa 903-0213, Japan.

[†]First Authors, *Corresponding Authors

Corresponding Authors

K.O., oka@tohoku.ac.jp, D.T., daitaki@cs.u-ryukyu.ac.jp

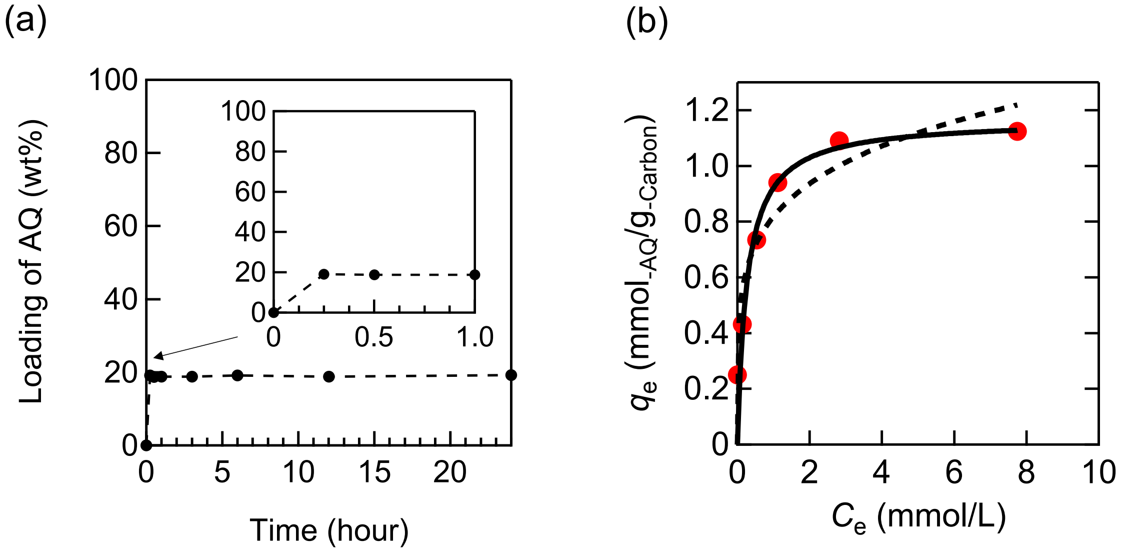

Figure S1. (a) AQ adsorption amount−time dependence. (b) Adsorption isotherms of AQ on AC with curve fitting to the Langmuir (solid line) and Freundlich models (dotted line).

The standard calibration curve was prepared by recording the absorbance values of various concentration of AQ at 335 nm. The suspension was magnetically stirred at room temperature for 24 h. The dispersion was centrifugated at 4000 rpm for 30 min, and the supernatant was analyzed using UV-vis spectroscopy.

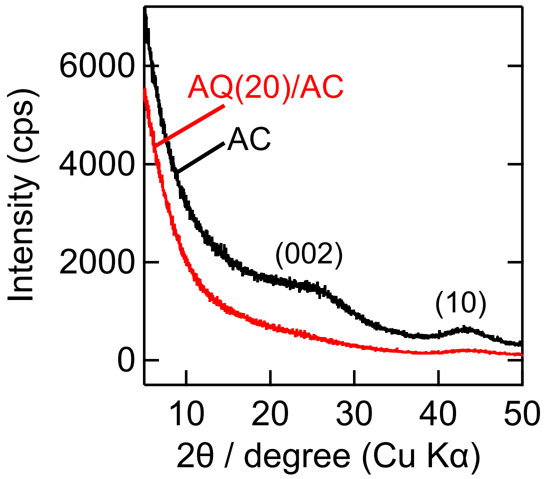

Figure S2. XRD pattern of AC (black) and AQ(20)/AC (red)

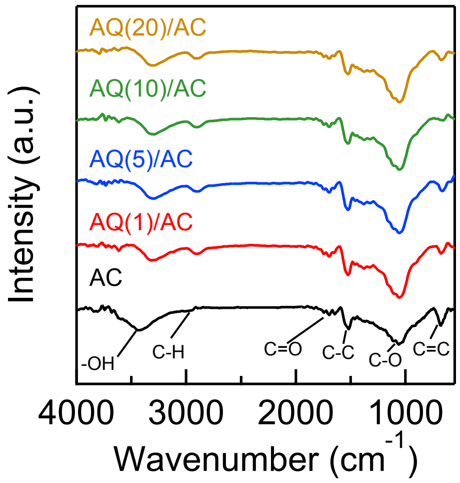

Figure S3. FT-IR spectra of AC (black), AQ(1)/AC (red), AQ(5)/AC (blue), AQ(10)/AC (green), and AQ(20)/AC (yellow).

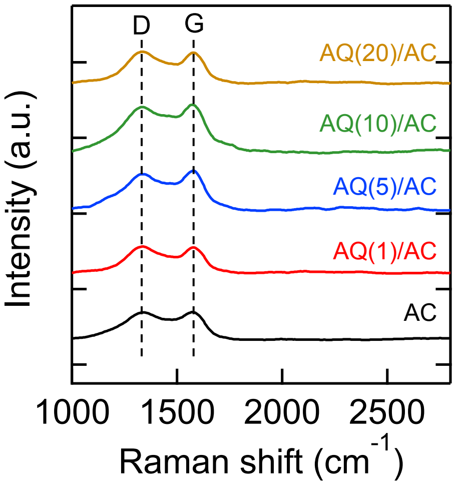

Figure S4. Raman spectra of AC (black), AQ(1)/AC (red), AQ(5)/AC (blue), AQ(10)/AC (green), and AQ(20)/AC (yellow).

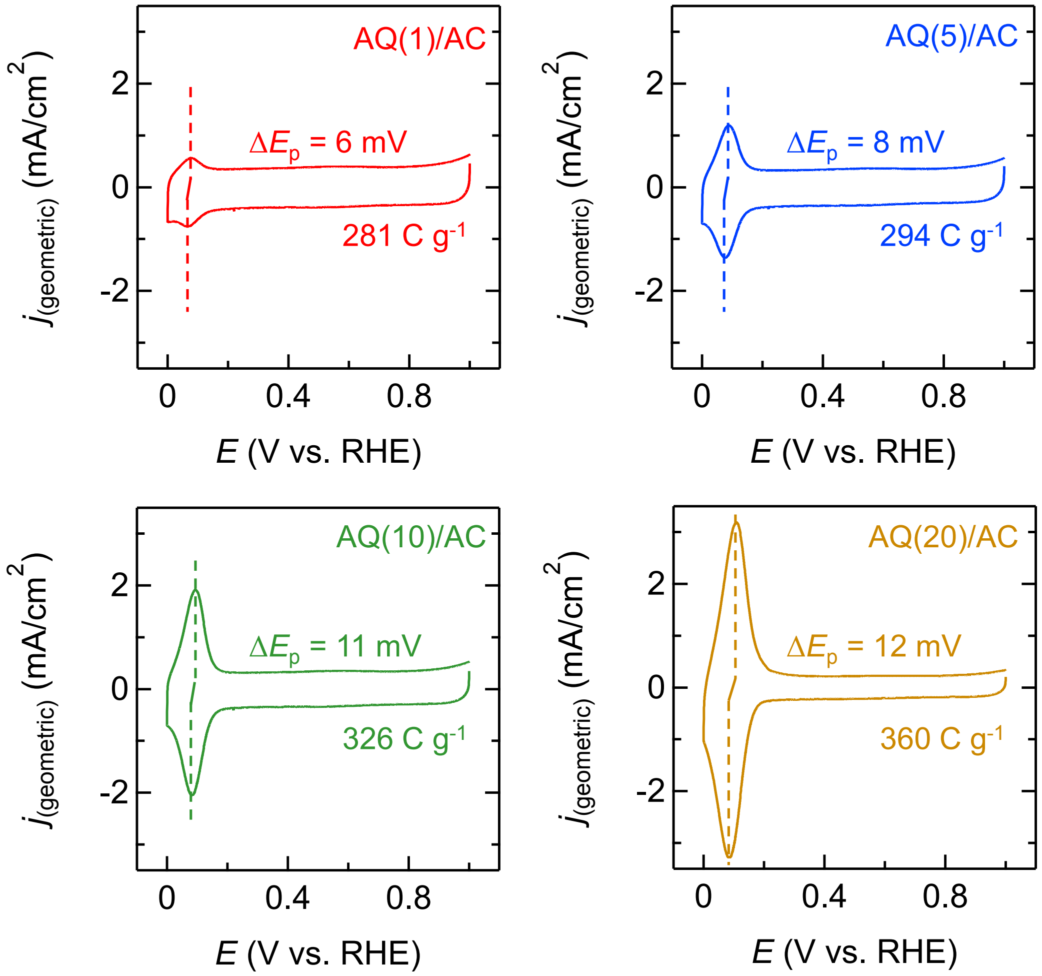

Figure S5. Cyclic voltammograms of AQ(1)/AC (red), AQ(5)/AC (blue), AQ(10)/AC (green), and AQ(20)/AC (yellow) at a scan rate of 5 mV s^{-1} in de-aerated 0.5 M H_2SO_4 (298 K).

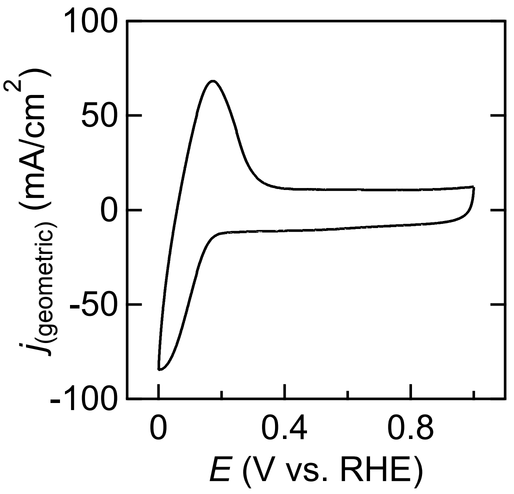

Figure S6. Cyclic voltammograms of AQ(20)/AC at a scan rate of 250 mV s^{-1} in de-aerated 0.5 M H_2SO_4 (298 K).

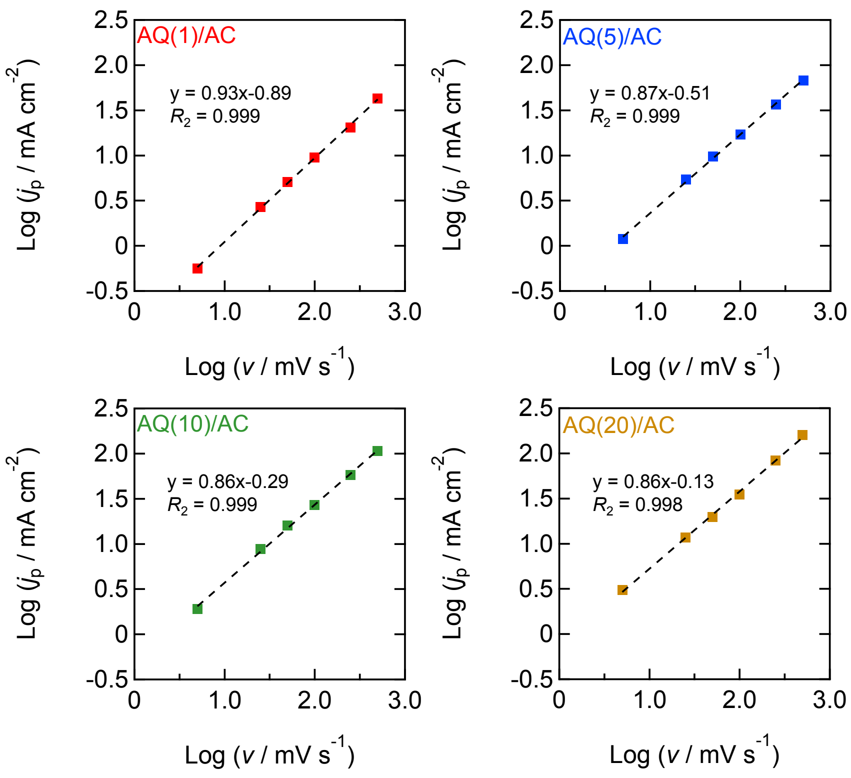

Figure S7. *b*-values (slopes) derived from the current peaks ($j_p = av^b$) of the cyclic voltammograms of AQ(1)/AC (red), AQ(5)/AC (blue), AQ(10)/AC (green), and AQ(20)/AC (yellow).

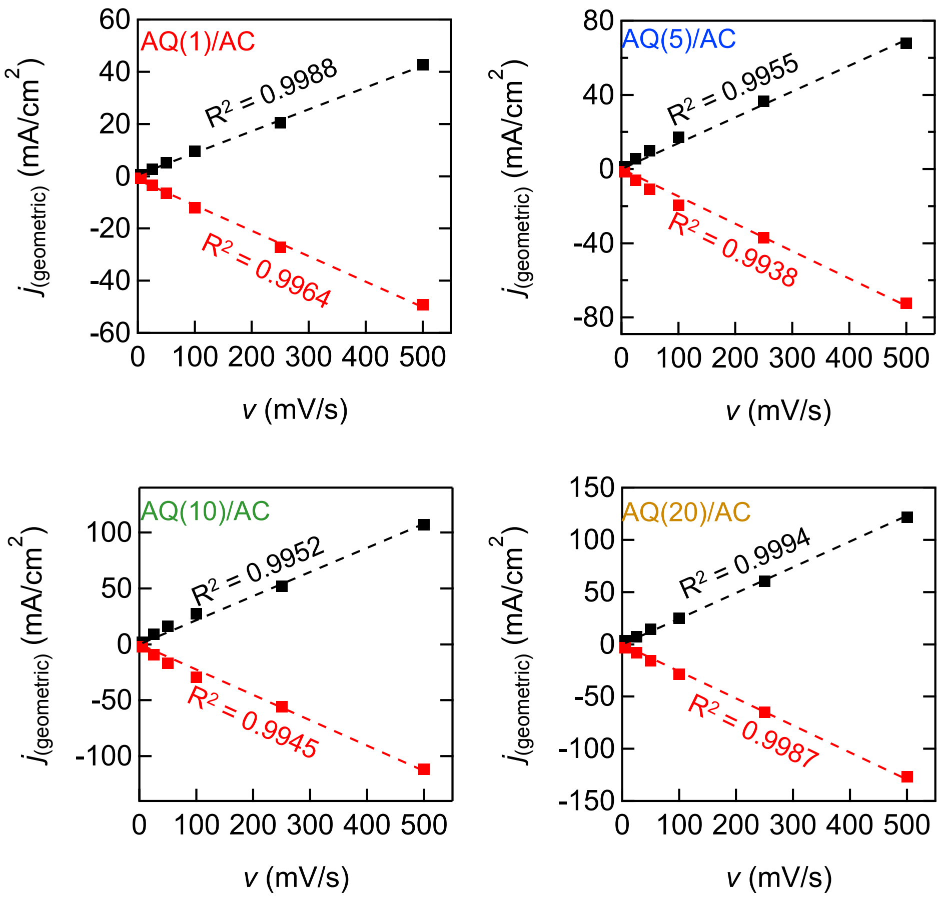

Figure S8. Changes in the anodic and cathodic peak currents density as a function of scan rate (v) of AQ(1)/AC (red), AQ(5)/AC (blue), AQ(10)/AC (green), and AQ(20)/AC (yellow).

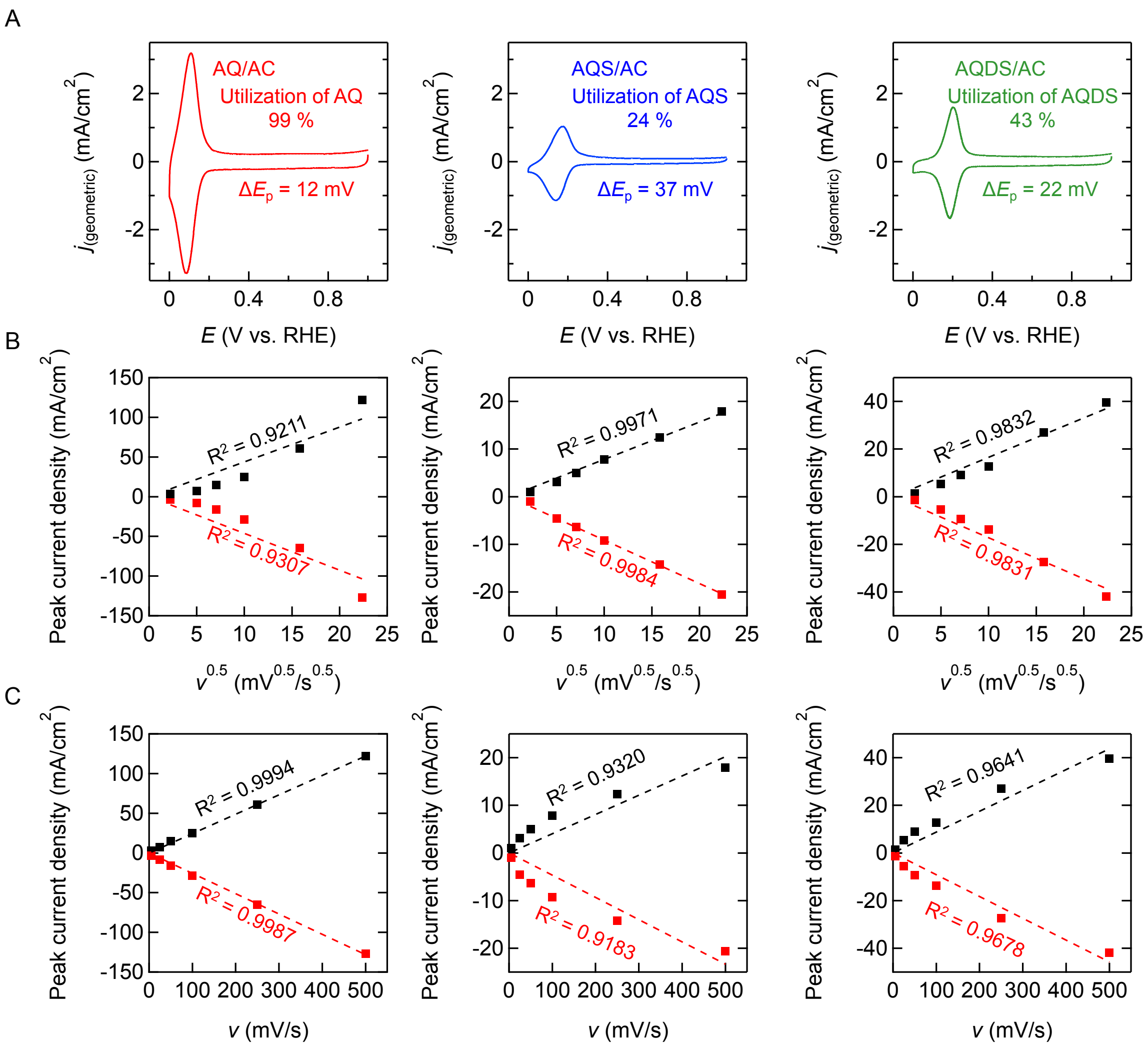

Figure S9. (A) Cyclic voltammograms at a scan rate of 5 mV s^{-1}, (B) changes in the anodic and cathodic peak currents density as a function of the square root of scan rate ($v^{0.5}$), and (C) changes in the anodic and cathodic peak currents density as a function of scan rate (v) for AQ/AC (red), AQS/AC (blue), and AQDS/AC (green) in de-aerated 0.5 M H_2SO_4 (298 K).

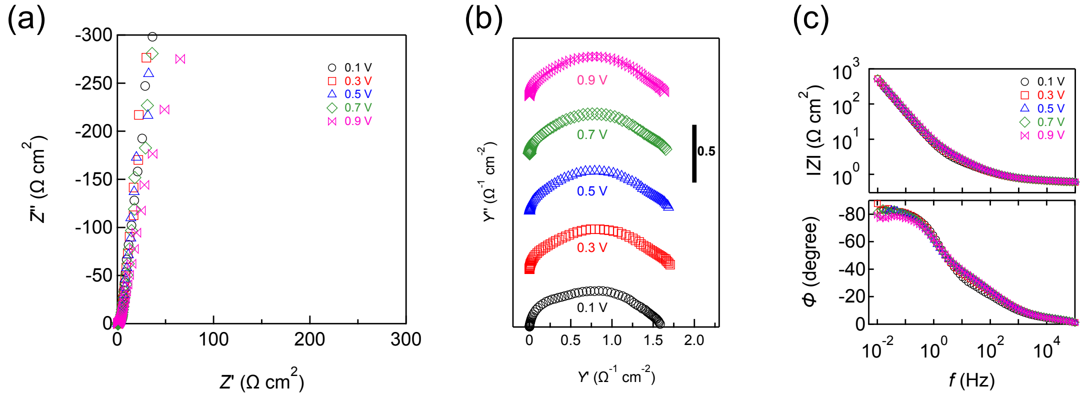

Figure S10. (a) Complex-plane impedance and (b) admittance plots at various electrode potentials of AC. (c) Frequency dependence of the magnitude |Z| and phase angle φ.

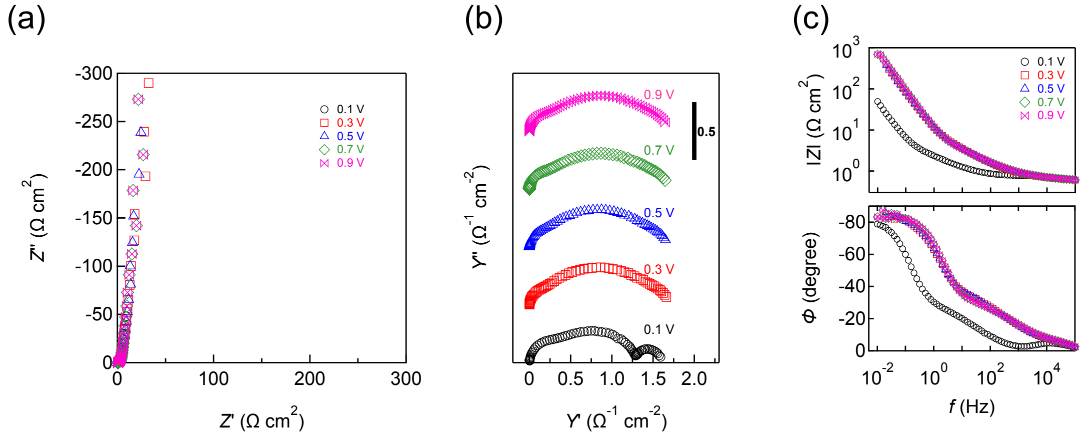

Figure S11. (a) Complex-plane impedance and (b) admittance plots at various electrode potentials of AQ(20)/AC. (c) Frequency dependence of the magnitude |Z| and phase angle φ.

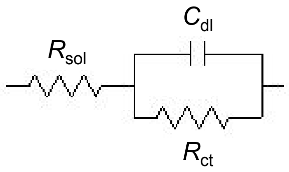

Figure S12. Equivalent circuits involving R_{sol} , R_{ct}, and C_{dl}.

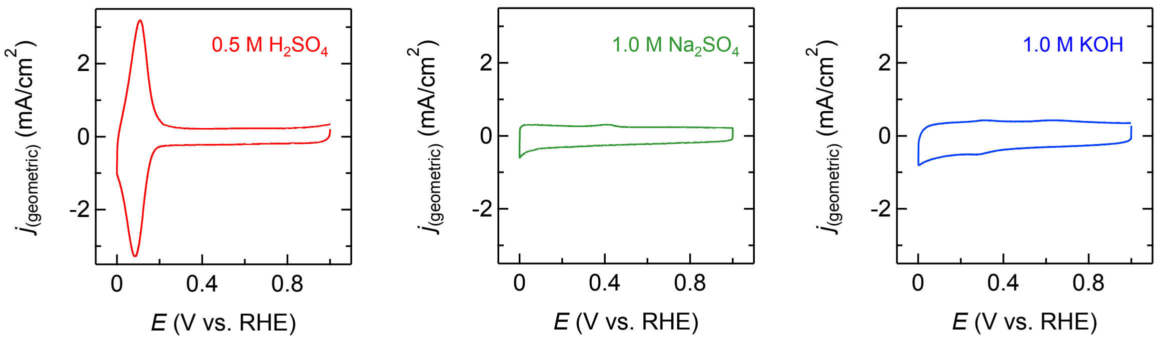

Figure S13. Cyclic voltammograms of AQ(20)/AC at a scan rate of 5 mV s^{-1} in de-aerated 0.5 M H_2SO_4 (red), 1.0 M Na_2SO_4 (green), and 1.0 M NaOH (blue).

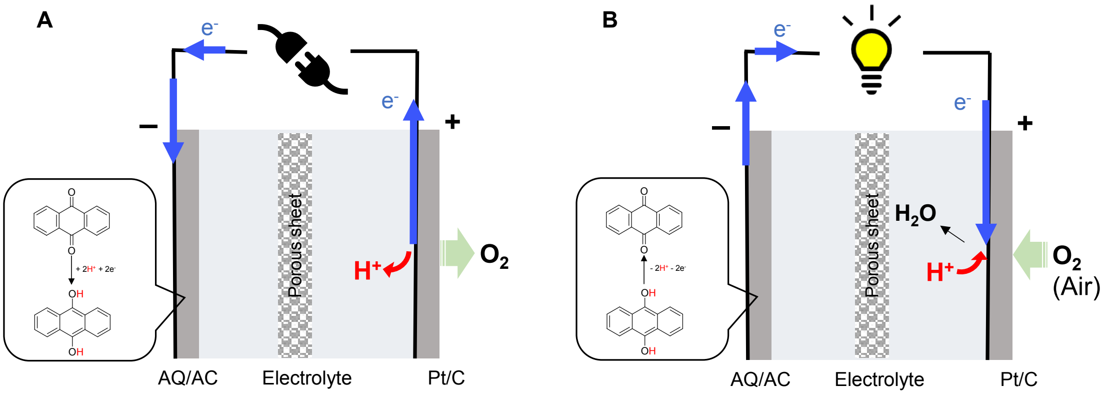

Figure S14. Schematic images of the battery for (A) charging and (B) discharging.

Table S1. Crystal data and structural refinement parameters of AQ.

Empirical formula	$C_{14}H_8O_2$
Formula weight	208.20
Temperature / K	173.15
Crystal system	monoclinic
Space group	$P2_1/c$
a / Å	7.8324(5)
b / Å	3.8879(2)
c / Å	15.6073(9)
α / °	90
β / °	102.544(6)
γ / °	90
Volume / $Å^3$	463.92(5)
Z	2
P_{calc} / g cm^{-3}	1.490
μ / mm^{-1}	0.100
F(000)	216.00
Crystal size / mm^3	0.391 × 0.07 × 0.04
Radiation	Mo Kα radiation (λ = 0.71075 Å)
2θrange for data collection / °	5.328 to 62.582
Index range	$-11 \leq h \leq 11$, $-4 \leq k \leq 5$, $-22 \leq l \leq 21$
Reflection collected	6202
Independent reflections	1435 [R_{int} = 0.0396, R_{sigma} = 0.0368]
Data/restraints/parameters	1435/0/73
Goodness-of-fit on F^2	1.099
Final *R* indexes [I>=2σ (I)]	R_1 = 0.0523, wR_2 = 0.1270
Final *R* indexes [all data]	R_1 = 0.0780, wR_2 = 0.1419
Largest diff. peak/hole / e$Å^{-3}$	0.24/–0.22

Table S2. D- and G-band intensity ratio (I_D/I_G) and area ratio (S_D/S_G) of AC and AQ/AC.

Sample	I_D/I_G	S_D/S_G
AC	0.99	1.37
AQ(1)/AC	1.03	1.41
AQ(5)/AC	0.92	1.29
AQ(10)/AC	0.95	1.33
AQ(20)/AC	1.04	1.42

Table S3. Specific surface area (S_{BET}), total pore volume (V_{total}), mesopore volume (V_{meso}), micropore volume (V_{micro}), and average micropore diameter of the carbon materials.

Samples	S_{BET} [a] (m^2 g^{-1})	V_{total} [b] (cm^3 g^{-1})	V_{meso} [c] (cm^3 g^{-1})	V_{mixro} [d] (cm^3 g^{-1})	$w_{ave.}$ [e] (nm)
AC	2002	0.94	0.11	0.83	0.90
AQ(20)/AC	1106	0.54	0.06	0.48	0.91

[a] Brunauer–Emmett–Teller (BET) surface area calculated at p/p_0 = 0.01–0.05
[b] Total pore volume estimated from amount adsorbed at p/p_0 = 0.96
[c] Mesopore volume calculated as $V_{total} - V_{micro}$
[d] Micropore volume calculated by the NLDFT method
[e] Average micropore width obtained from the NLDFT method

Table S4. Summary of previous results, electrode details, and reaction conditions.

Carbon	Molecule	Electrolyte	v (mV s^{-1})	ΔE_p (mV)	Ref.
Carbon black (Black Pearls 2000)	Anthraquinone	0.1 M H_2SO_4	50	~50	3
Ketjen black	2,5-Dichloro-1,4 benzoquinone	1 M H_2SO_4	1	~50	7
Hierarchical porous graphitic carbon fibers	Anthraquinone	1 M H_2SO_4	10	~30	8
Reduced graphene oxide	Benz[a]anthracene 7,12-quinone and 2,5 dihydroxy-p benzoquinone	1 M H_2SO_4	10	~20	15
Reduced graphene oxide nanosheet	Anthraquinone monosulfonate	0.1 M phosphate buffer	10	~100	16
Hierarchical porous carbon nanotubes	Anthraquinone	1 M H_2SO_4	10	46	17
Carbon black (N330)	Sesamol	Phosphate buffer solution	100	63	18
Carbon black (Vulcan XC 72)	Anthraquinone	1 M H_2SO_4	100	~50	19
Activated carbon (Supercap BP10)	1,4-Benzoquinone	0.5 M H_2SO_4	10	~50	20
Diamond substrate	[Ru(tpy)2]2+	1 M $HClO_4$	1	~90	21

Table S5. Specific capacitance of AC and AQ/AC at different scan rate.

Scan rate ($mV\ s^{-1}$)	AC ($C\ g^{-1}$)	AQ(1.0)/AC ($C\ g^{-1}$)	AQ(4.9)/AC ($C\ g^{-1}$)	AQ(8.3)/AC ($C\ g^{-1}$)	AQ(19.0)/AC ($C\ g^{-1}$)
5	221	231	265	294	359
25	194	214	243	276	319
50	180	205	233	266	293
100	161	196	218	250	255

Table S6. Capacity rate of AQ/AC.

Sample	EDLC (%)	Pseudo-capacitance (%)
AQ(1)/AC	95.9	4.1
AQ(5)/AC	82.7	17.3
AQ(10)/AC	74.0	26.0
AQ(20)/AC	51.2	48.8

Table S7. Utilization of AQ in active materials.

	AC	AQ(1)/AC	AQ(5)/AC	AQ(10)/AC	AQ(20)/AC
Specific capacity (C g^{-1})	221	231	265	294	360
Amount of adsorbed quinone by UV-Vis (mmol/$g_{-Carbon}$)	–	0.05	0.250	0.432	1.124
Amount of electrochemical active AQ (mmol/$g_{-Carbon}$)	–	0.048	0.246	0.428	1.119
Utilization rate of AQ (%)	_	99.7	99.9	99.9	99.9

Table S8. The summary of the performance of rechargeable organic-based aqueous air batteries.

Material	Utilization of quinone (%)	Capacity retention (%)	Reference
AQ/AC	100.0	99	This work
NHCC	59.7	99	27
P14AQ	97.3	95	28
BBL	83.1	99	29
pEP(NQ)E	100.0	98	30
AQ-CMP	90.7	99	31
TpOMe-DAQ	96.1	99	32
PVAQ	93.4	87	33
PDBM	98.9	97	34
